# Juvenile systemic lupus erythematosus presenting as pancarditis

**DOI:** 10.1186/s12969-019-0372-z

**Published:** 2019-11-04

**Authors:** D. O’Leary, C. O’Connor, L. Nertney, E. J. MacDermott, D. Mullane, O. Franklin, O. G. Killeen

**Affiliations:** 1 National Centre for Paediatric Rheumatology, CHI at Crumlin, Dublin, Ireland; 2 School of Medicine, University College Dublin, Dublin, Ireland; 3 Department of Paediatrics, Cork University Hospital, Cork, Ireland; 4 Department of Paediatric Cardiology, CHI at Crumlin, Dublin, Ireland

**Keywords:** Juvenile, SLE, Pancarditis

## Abstract

**Background:**

Systemic lupus erythematosus (SLE) is a chronic autoimmune disease with marked variation in its clinical presentation. Juvenile SLE (jSLE) accounts for 15–20% of all cases and is diagnosed when SLE manifests before 18 years of age. Pancarditis is a rare complication of SLE, regardless of age of disease onset.

**Case presentation:**

We report a case of jSLE in a 15 year old Caucasian female presenting with an acute episode of pancarditis and multiorgan dysfunction who was successfully treated with systemic corticosteroids and cyclophosphamide.

**Conclusion:**

Pancarditis can be a presenting feature of jSLE which was previously unreported. A high index of suspicion for severe cardiac involvement is required at all stages of disease.

## Background

Juvenile systemic lupus erythematosus (jSLE) is defined as systemic lupus erythematosus (SLE) with onset before 18 years of age and accounts for 15% of SLE patients [1]. The annual incidence of jSLE is estimated to be 0.3–0.9/100,000 and is generally lower in Caucasian children [2, 3]. Juvenile SLE is known to be associated with a higher incidence of arthritis, nephritis, haematologic and neurologic manifestations than that seen in adult-onset disease [2]. In particular, adolescent-onset SLE is associated with more aggressive disease [1]. Fifty percent of juvenile SLE patients present in adolescence [2].

Overall, less than 10% of jSLE patients have severe cardiorespiratory involvement at presentation [3]. Pancarditis has never been reported as a presenting feature in jSLE. Pancarditis involves inflammation of the pericardium, myocardium and endocardium and may present acutely with congestive cardiac failure or sudden death [4, 5]. In the setting of SLE, pancarditis may respond well to treatment with systemic corticosteroids which makes timely recognition important [6].

## Case presentation

A 15 year old Caucasian female was transferred from a secondary care paediatric unit. She presented with a two-day history of progressive dyspnoea, cough and palpitations on a background of recent onset arthralgia, alopecia and oral ulceration. Clinical examination revealed hypertension (blood pressure 170/110 mmHg), pallor with a malar rash, symmetrical polyarthritis of the interphalangeal and metacarpophalangeal joints, alopecia and oral ulceration.

Investigations revealed normocytic anaemia, haemoglobin 95 g/l (normal 120-160 g/l), lymphopaenia, lymphocytes 0.9 × 109/l (normal 1.2–5.2 × 109/l)), elevated inflammatory markers with an erythrocyte sedimentation rate (ESR) of 77 mm/hr. (normal 1-9 mm/hr) and c-reactive protein (CRP) of 38 mg/l (normal < 10 mg/l) and moderately impaired renal function with urea 14.4 mmol/l (normal 2.0–6.0 mmol/l), creatinine 154 μmol/l (normal 30-90 μmol/l). Coagulation screen showed a slightly prolonged prothrombin time (PT) of 13 s (normal 10.2–12.0 s) but was otherwise normal. Albumin was low (28 g/l, normal 36-50 g/l) and liver function tests were normal. Microscopic haematuria and proteinuria were present with an elevated urine albumin:creatinine ratio of 1217 mg/mmol (normal < 3.4 mg/mmol). Antinuclear antibody titres were strongly positive with a titre of 1:160, speckled pattern. Anti double-stranded DNA was positive with a titres of > 379 IU/ml (normal 0-10 IU/ml) and positive Crithidia assay >/= 1:160. Anti-Smith and anti-RNP antibodies were both positive with titres of > 480 U/ml (normal 0–5.0 U/ml) and > 240 U/ml (normal 0-5 U/ml) respectively. There was marked hypocomplementaemia with C3 0.44 g/l (normal 0.7–1.7 g/l), C4 0.06 g/l (normal 0.1–0.7 g/l) and absent CH100 classical and alternative pathway components. Antiphospholipid, anti-SSA and anti-SSB antibodies were all negative. Chest x-ray showed bilateral pleural effusions and cardiomegaly with a cardiothoracic ratio of 0.67. Initial echocardiography showed a large pericardial effusion with diastolic compression of the right atrium and ventricle suggestive of cardiac tamponade. The left ventricle was dilated with an ejection fraction of 25% and there was mild mitral, tricuspid and aortic valvular regurgitation. Treatment was commenced with high-dose intravenous methylprednisolone (30 mg/kg/dose, maximum dose of 1 g) and diuretics and immediate transfer to a tertiary paediatric intensive care unit was arranged.

**Fig. 1:**
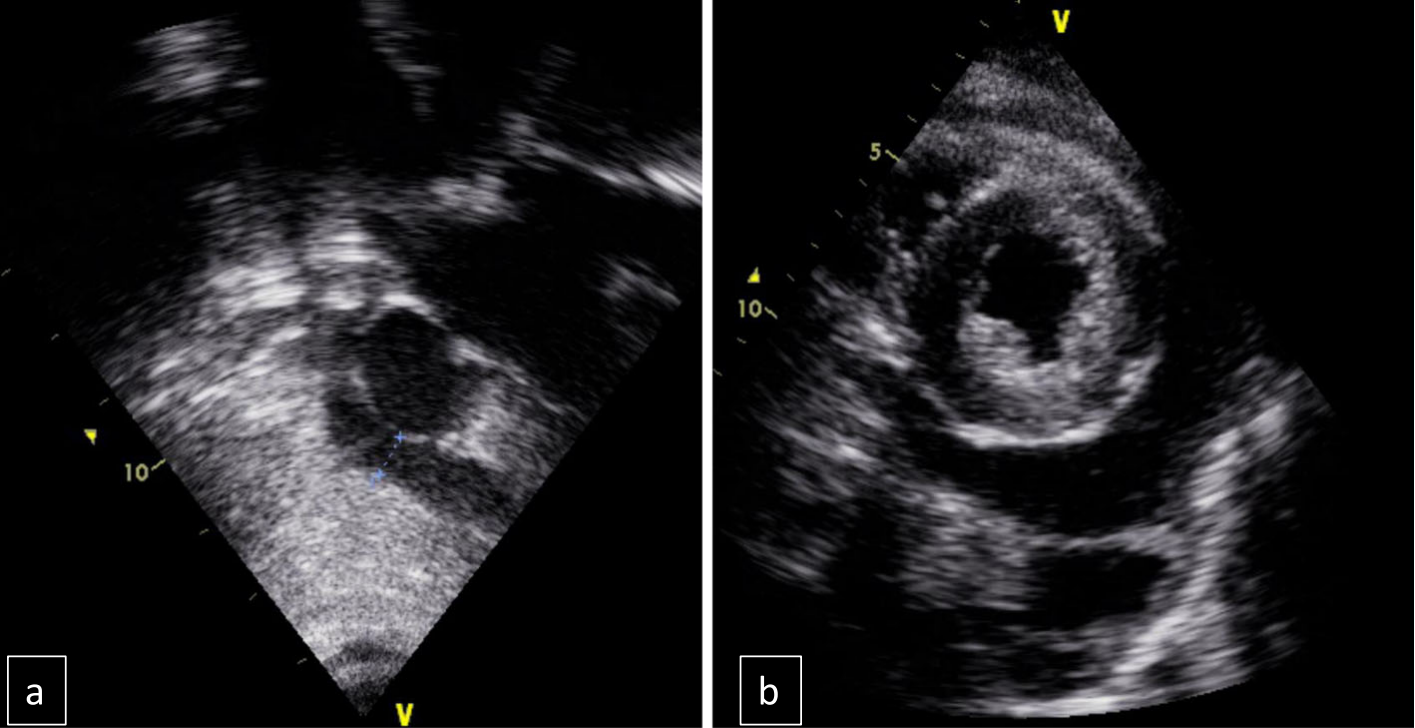
Echocardiography on admission to intensive care. **a**: pericardial effusion behind the right atrium. **b**: parasternal short axis view with a pericardial effusion

On admission to the intensive care unit she had developed periorbital oedema and ascites with worsening dyspnoea and reduced oxygen saturation. Echocardiography revealed a large pericardial effusion, oedematous myocardium and valvulitis with an ejection fraction of 13% with no evidence of tamponade (see Fig. 1). Renal function deteriorated further with a creatinine increase to 270 μmol/l (normal range 30-90 μmol/l) and the patient became anuric. Intermittent positive pressure ventilation, inotropic support, plasma exchange and haemodialysis were required. High-dose intravenous methylprednisolone was continued for 3 days and then changed to oral prednisolone at 1 g/kg/day. Cyclophosphamide was commenced at a dose of 850 mg/m2 on day four of admission due to severe renal impairment and ongoing need for haemodialysis and multiorgan involvement.

Follow-up echocardiography showed normalisation of function by day five of admission with a small pericardial effusion as the only persistent abnormality. Renal biopsy revealed grade 4 lupus nephritis. The patient was discharged from the intensive care unit on day seven of admission and subsequently discharged from the hospital on day fourteen. Treatment at discharge included a weaning dose of prednisolone, hydroxychloroquine, enalapril and carvedilol. Cyclophosphamide treatment was continued monthly for a total of six doses after which the patient was maintained on further immunosuppression. Remission has been maintained with mycophenolate mofetil and hydroxychloroquine over the past 2 years.

## Discussion

The reported rate of cardiac involvement in jSLE varies widely [7]. Pericarditis is the most common cardiac manifestation of jSLE, occurring in up to 25% of patients [8, 9]. Myocarditis and non-infective endocarditis are seen less frequently [10, 11] but can be severe and life-threatening. Cardiac tamponade is infrequently reported in SLE regardless of age of onset [8, 12]. Pancarditis involves inflammation of the pericardium, myocardium and endocardium. It is a rare but recognised complication of SLE [6, 12]. It has been described as a presenting feature of adult-onset SLE in two case reports [4, 13]. The first patient was antiphospholipid antibody positive, which is associated with an increased risk of cardiac involvement [6, 8, 13]. In this case the patient responded to systemic steroid treatment with resolution of both pericarditis and endocarditis and almost complete return to normal left ventricular function. A similar response has been reported in other SLE patients who developed pancarditis later in the disease course [6]. In the second case, the diagnosis of lupus pancarditis was made on autopsy [4]. Both patients had clinical features consistent with SLE in other organ systems at the time of diagnosis.

Transthoracic echocardiography is a widely accepted tool for the diagnosis of endocarditis and pericarditis [14]. Endomyocardial biopsy is considered the gold standard for diagnosis of myocarditis because it is the only investigation that identifies the underlying aetiology [15]. With recent advances in non-invasive diagnostic techniques, biopsy is performed less frequently. However, it may still be required in unexplained or refractory cases [16]. In this patient there were clear signs of myocarditis on echocardiography and the underlying aetiology was clinically apparent as SLE. In addition, myocardial abnormalities responded quickly to systemic steroid treatment. Therefore, further investigation with either cardiac MRI or endomyocardial biopsy was not indicated in this patient.

Antiphospholipid antibodies and anti-SSA/SSB antibodies are associated with cardiac involvement in SLE [6, 8]. Both antibodies were negative in this patient. A small case series of predominantly Caucasian patients with jSLE demonstrated an association between anti-Smith and anti-RNP antibodies and cardiac involvement [7], both of which were present in this patient.

## Conclusions

This is the first reported case of jSLE presenting with pancarditis. There are no guidelines for the treatment of this rare complication of SLE. The use of systemic steroids in this case is supported by reports on adult patients with SLE-related pancarditis. Subsequent immunosuppressive treatment was primarily directed by the degree of renal involvement. Pancarditis represents a severe cardiac complication with potential fatal outcome. This complication should be considered in acutely unwell patients with SLE regardless of age or disease stage.

## Data Availability

Data sharing not applicable to this article as no datasets were generated or analysed during the current study.

## Abbreviations

CRP: C-reactive protein
ESR: Erythrocyte sedimentation rate
jSLE: Juvenile systemic lupus erythematosus
SLE: Systemic lupus erythematosus
